# Effects of Saccadic Bilateral Eye Movements on Episodic and Semantic Autobiographical Memory Fluency

**DOI:** 10.3389/fnhum.2013.00630

**Published:** 2013-09-26

**Authors:** Andrew Parker, Adam Parkin, Neil Dagnall

**Affiliations:** ^1^Department of Psychology, Manchester Metropolitan University, Manchester, UK

**Keywords:** bilateral eye movements, autobiographical memory, episodic memory, semantic memory, memory fluency, hemispheric interaction

## Abstract

Performing a sequence of fast saccadic horizontal eye movements has been shown to facilitate performance on a range of cognitive tasks, including the retrieval of episodic memories. One explanation for these effects is based on the hypothesis that saccadic eye movements increase hemispheric interaction, and that such interactions are important for particular types of memory. The aim of the current research was to assess the effect of horizontal saccadic eye movements on the retrieval of both episodic autobiographical memory (event/incident based memory) and semantic autobiographical memory (fact based memory) over recent and more distant time periods. It was found that saccadic eye movements facilitated the retrieval of episodic autobiographical memories (over all time periods) but not semantic autobiographical memories. In addition, eye movements did not enhance the retrieval of non-autobiographical semantic memory. This finding illustrates a dissociation between the episodic and semantic characteristics of personal memory and is considered within the context of hemispheric contributions to episodic memory performance.

## Introduction

Autobiographical memory refers to a range of stored information and knowledge pertaining to the self, and thus a form of personal memory. It is considered to comprise of both episodic (event and instance specific) and semantic (general self knowledge and facts about oneself) components (Conway, 1990, 2005; Dritschel et al., 1992; Conway and Pleydell-Pearce, 2000; Levine, 2004). The experiment presented here is concerned with the influence of saccadic bilateral (horizontal) eye movements on the retrieval of episodic and semantic components of autobiographical memory.

The reason for the interest in saccadic bilateral eye movements relates to a broader research literature regarding the role of hemispheric interaction in memory. It has been proposed that episodic memory processing is, in part, dependent upon hemispheric interactions (e.g., Christman et al., 2003; Habib et al., 2003; Christman and Propper, 2010). The importance of cerebral interaction in episodic memory comes from a range of findings. For example, research with commissurotomized patients or those with callosal agenesis show impaired performance on free recall and recognition, both of which are episodic memory tests (Phelps et al., 1991; Geffen et al., 1994; Cronin-Golomb et al., 1996; Jha et al., 1997).

Personal handedness has also been used as a behavioral marker for hemispheric interaction as several studies have found that mixed or inconsistent handed individuals have a relatively larger corpus callosum compared to right-handed individuals (e.g., Witelson, 1985; Denenberg et al., 1991; Habib et al., 1991; Clarke and Zaidel, 1994). The larger size of the corpus callosum subsequently provides a basis for greater hemispheric interaction (Christman, 1993, 1995; Niebauer et al., 2002). Results have revealed that mixed-handed individuals outperform those who are strongly right-handed on a range of episodic memory tasks including: free recall following incidental and intentional learning (Propper et al., 2005; Christman and Butler, 2011), enhanced remember responses in the remember-know paradigm (Propper and Christman, 2004), more accurate source/associative memory (Lyle et al., 2008b, 2012; Chu et al., 2012), and more successful learning of a foreign vocabulary (Kempe et al., 2009).

Research using neuroimaging procedures have also revealed interesting findings regarding the role of hemispheric interaction. Observations that the left and right prefrontal regions appeared to be preferentially activated during the encoding (vs. retrieval) of episodic information led to the proposal of the Hemispheric Encoding and Retrieval Asymmetry (HERA) model (Nyberg et al., 1996; Habib et al., 2003). This model essentially describes a functional asymmetry between the left and right hemispheres in terms of cognitive operations that are independent of the type of material being processed. A number of studies have found broad support for this idea using a range of techniques including PET (Tulving et al., 1994), fMRI (McDermott et al., 1999; Kompus et al., 2011), EEG (Babiloni et al., 2004, 2006), ERPs (Düzel et al., 1999), and TMS (Rossi et al., 2004, 2006; Gagnon et al., 2010; Manenti et al., 2011). In spite of the evidence in favor of the HERA model, not all research is equally supportive. For example, it has been argued that the main determinant of asymmetries is related to material-specific processing biases (e.g., Wagner et al., 1998; Miller et al., 2002) (but see Habib et al., 2003 for counter-arguments). Also, a case has been made for the hypothesis that when retrieval is effortful or demanding of resources, then retrieval itself elicits bilateral activations (Nolde et al., 1998). In some respects, Nolde et al. (1998) extend the HERA model by continuing to emphasize left hemisphere activity during encoding but either right or bilateral activity during retrieval depending on the complexity of the retrieval task. Despite some problems with HERA, it has been contended that successful performance on tasks of episodic memory are dependent upon the interaction between right hemisphere based retrieval processes operating upon the products of left hemisphere based encoding processes (Christman et al., 2003; Christman and Propper, 2010). Even if effortful retrieval involves bilateral activations, then presumably, successful interaction between the hemispheres would be important for retrieval success.

One particularly relevant avenue of research has focused on the effects of saccadic bilateral eye movements on memory. A number of experiments have demonstrated that episodic memory can be improved if retrieval is preceded by a period of 30 s of saccadic bilateral eye movements. For example, Christman et al. (2003), presented subjects with a list of words during the encoding phase of an experiment. This was followed by 30 s of bilateral eye movements prior to a test of episodic recognition memory. Compared to a range of control conditions, it was found that bilateral saccades improved overall memory accuracy. In addition, and consistent with predictions, bilateral enhancement was absent on a non-episodic test of implicit memory.

This and similar findings have been observed across a number of laboratory-based tests assessing different aspects of episodic memory such as: associative recognition and context memory (Parker et al., 2008; Lyle et al., 2012), visual scenes (Parker et al., 2009; Lyle and Jacobs, 2010), landmark shape and spatial location information (Brunyé et al., 2009), and free recall of neutral and emotional words (Samara et al., 2011; Nieuwenhuis et al., 2013).

Eye movements have also been shown to reduce false memory in paradigms that are designed to elicit high proportions of false memories, such as the Deese-Roediger-McDermott (DRM) paradigm (Christman et al., 2004; Parker and Dagnall, 2007). As a result, eye movements can be shown to increase memory accuracy by both increasing the hit rate and reducing the false alarm rate (e.g., Lyle et al., 2008a; Parker et al., 2008; Brunyé et al., 2009). Interestingly, a dissociation between eye movements and memory type has been found in one experiment. Christman et al. (2003) compared the effects of eye movements on performance on tests of both episodic recognition and the perceptual implicit test of word-fragment completion (WFC). Previous research has demonstrated that WFC is little influenced by encoding tasks that encourage meaning based analyses of the stimuli (e.g., Roediger et al., 1992). However, WFC is influenced by changes in perceptual characteristics of items between study and test (e.g., Blum and Yonelinas, 2001) and is largely considered to be dependent upon perceptual-lexical processing (e.g., Nelson et al., 1989; Richardson-Klavehn and Gardiner, 1998). The results of Christman et al. (2003) found that saccadic bilateral eye movements influenced episodic recognition only.

One explanation for these findings is that saccadic bilateral eye movements bring about increased hemispheric interaction. In particular, lateral eye movements have been found to be associated with activations in the contralateral hemisphere (Dean et al., 2004; Kastner et al., 2007). Consequently, performing a sequence of right-left eye movements is assumed to result in bihemispheric activation. This in turn leads to equalized activation between the two hemispheres and increased hemispheric interaction (Christman et al., 2003; Christman and Propper, 2010). Potentially therefore, this forms the foundation for superior episodic memory, by providing a basis for augmented encoding-retrieval interactions.

Both handedness and eye movement effects have been shown to extend beyond memories acquired in the laboratory and improve the recall of autobiographical information. For example, Propper et al. (2005) found that mixed-handed individuals recalled more actual (true) autobiographical events and were less prone to false recall. Subsequently, Christman et al. (2006) established both that mixed-handedness and bilateral saccades enhanced the recall of earlier childhood memories. Later, Parker and Dagnall (2010) furthered this work, observing similar enhancing effects of handedness and eye movements on the retrieval of recollective (and hence episodic) qualities associated with autobiographical remembering.

Thus far, research on autobiographical memory and saccadic eye movements (and handedness) has been limited to episodic autobiographical memory. As noted earlier, autobiographical memory consists of both episodic and semantic components. Episodic autobiographical memory refers to memory for event-specific details within a spatio-temporal context. Semantic autobiographical memory in contrast refers to personal facts and knowledge about the self and life experiences (Conway, 2005; Kopelman et al., 1989; Tulving, 2002). Research indicates that episodic and semantic components of autobiographical memory can be dissociated as a function of a range of factors such as: brain damage (e.g., Kopelman et al., 1989; Murphy et al., 2008; Coste et al., 2011), dementia (e.g., Greene et al., 1995; Smith et al., 2010), and aging (e.g., Levine et al., 2002; Piolino et al., 2002). Patterns of autobiographical memory dysfunction are also associated with cortical-specific changes in cell loss/damage (Gilboa et al., 2005).

The current research extends previous findings by considering the effects of eye movements on both episodic and semantic personal memory. To achieve this, it employs an autobiographical fluency task that enables both components to be measured on a common metric (Dritschel et al., 1992). In this test, participants are required to produce aloud as many exemplars as possible to a set of categories covering episodic and semantic personal memory (and general semantic memory as an additional point of comparison). The measure of interest is the number of exemplars produced per unit of time over 90 s (i.e., fluency). This technique (and similar fluency measures) has been employed in a variety of studies to assess the episodic and semantic aspects of autobiographical memory (e.g., Dritschel et al., 1992; Greene et al., 1995; Matuszewski et al., 2009; Smith et al., 2010; Coste et al., 2011; Unsworth et al., 2012).

In the current experiment, memory was assessed after a pre-task activity phase that involved either bilateral, vertical, or no-eye movements. A vertical condition was employed similar to previous work (e.g., Christman et al., 2003; Parker and Dagnall, 2007; Brunyé et al., 2009). The purpose of the vertical condition was to act as an additional control to assess the specificity of eye movement effects as opposed to influences due to increased oculo-motor activity. According to Christman et al. (2003), finding effects specific to the horizontal condition is supportive of the importance of hemispheric interaction.

In the current experiment, the pre-task activity was repeated prior to the recall of each memory type. The reason for this relates to the likelihood that eye movement effects on cognition are likely to be time-limited. Although this is a reasonable idea, we are not aware of any specific experiments that have directly addresses this notion. However, some research does have some bearing upon the issue. For example, Brunyé et al. (2009) argued that the duration of the effects might be similar to the duration of increased neural excitability brought about by cortical transcranial magnetic stimulation that on average is up to about 4 min (Pasqual-Leone et al., 1994). In addition, in a test of divergent thinking, Shobe et al. (2009) found that eye movement effects lasted up to 3 or 9 min depending on the measure used. Consequently, it is considered that the influence of eye movements is limited to a particular time-window, although the precise duration of this window in relation to memory retrieval is not yet known.

Based upon previous research and ideas of Christman et al. (2003), it is hypothesized that bilateral saccades will facilitate the retrieval of episodic autobiographical memory. It is expected that both forms of semantic memory will be uninfluenced. Thus episodic and semantic memories are expected to dissociate as a function of bilateral saccades.

## Materials and Methods

### Design

The experiment had three independent variables and formed a three (eye movement condition; bilateral vs. vertical vs. no-eye movement) between-subjects by two (autobiographical memory period; age 5–11 vs. 12–18) within-subjects by three (recall period; 30 vs. 60 vs. 90 s) within-subjects mixed ANOVA. The dependent variable was the cumulative number of memories recalled in each of the conditions. In particular, the number of episodic memories, friends names, teachers names, and category examples.

### Participants

A total of 69 participants took part in the experiment study and were aged between 18 and 37 years. A total of 23 were assigned randomly to each of the eye movement conditions. The mean ages of the participants within each condition were 22.96 for the bilateral group, 22.91 for the vertical group, and 23.13 for the no-eye movement group. They were recruited from both inside and outside the university campus building and took part voluntarily after providing informed consent.

### Materials and apparatus

Test booklets were prepared that consisted of two main sections. The first section allowed for the recording of participant information. The second section contained the experimental instructions pertaining to the recall of episodic autobiographical memory, semantic autobiographical memory, and general semantic memory. With the exception of general semantic memory, each of the aforementioned was further subdivided into autobiographical memory periods covering memories from 5 to 11 and 12 to 18 years. The test booklets contained the experimental instructions and were later used to record the memory scores of the subjects across each of the conditions.

A digital timer was used to time the 90 s given for each memory recall test. A Dictaphone was used to record the memories produced by the subjects. This allowed the experimenter to assess memory recalls over the time period and remove any repeated responses from the participant recall protocol.

Following previous similar work, a computer program was designed to initiate eye movements. This was done by flashing a black circle against a white background from side to side (bilateral condition), up and down (vertical condition), or on and off in the center of the screen (fixation condition). The circle moved (flashed) once every 500 ms and in the eye movement conditions was located approximately 27°of visual angle apart. The average size of the computer monitor was 55 cm (diagonal) and viewing distance as adjusted to maintain 27°of visual angle.

### Procedure

All participants were tested individually and randomly assigned to the eye movement conditions. Initially, each participant was provided with the test booklet and asked to fill in the section requesting personal details. Once this had been done, the booklet was taken from the participant and retained by the experimenter.

The experimenter then requested the participant to face a computer monitor and was told that the next part of the experiment would begin. They were informed that this would consist of a repeating cycle of four phases in which they would be asked to view a moving (or stationary) dot on a screen followed by a test of memory. All instructions were presented aurally.

For those allocated to the bilateral (vs. vertical) condition, the instructions requested subjects to follow the moving dot from side to side (vs. up and down) by making horizontal (vs. vertical) eye movements. In the no-eye movement condition, subjects were asked to fixate their attention on the dot flashing on and off in the center of the screen. The experimenter monitored compliance with these instructions. After 30 s of the allotted condition, the memory test began. This procedure was repeated a total of four times, once prior to each recall test.

The standardized instructions (based on Dritschel et al., 1992) were read aloud by the experimenter to the participant according to the type of memory tested (specific personal events (episodic autobiographical), friends names (semantic autobiographical), teachers names (semantic autobiographical), and category examples (general semantic).

For episodic autobiographical memory the instructions read “For this test, I would like you to recall as many personal memories of events from two periods in your life. The first period is between 5 and 11 years old and the second period is between 12 and 18 years old. For each of these periods I would like you to recall as many memories as you can within 90 s. Please try to name specific event memories, such as “the time I beat my best friend in the school swimming competition” rather than general memories, such as “having a paper round.” Please do not go into describe in detail about each memory, just state each one as it comes to mind and then move onto the next. Participants were informed that they did not have to disclose any memories they were not comfortable with recalling or sharing.

For semantic autobiographical memory the instructions read “For this test, I would like you to recall as many autobiographical facts as you can from two periods in your life. The first period is between 5 and 11 years old and the second period is between 12 and 18 years old. For each of these periods I would like you to recall as many autobiographical facts as you can within 90 s. By autobiographical facts, in this case I mean names of school friends (vs. teachers). You do not need to tell me each memory in detail, just try to recall as many facts as you can about your life.”

For general semantic memory the instructions read “For this test, I would like you to generate as many examples from two semantic categories as you can. I will give you 90 s to generate from each semantic category. By generating examples from semantic categories what I mean is this, if I were to say transport then I would like you to say as many examples of transport that you can such as cars, trains, boats ships, etc. Just state out loud the examples that come to mind. You do not need to tell me each example in detail, just try to generate as many examples as you can.” After presenting these instructions, the experimenter read aloud either animals or vegetables in a randomized order and the recall period commenced. Once the recall period had expired, the next category was presented and the second recall period for semantic memory commenced.

Once the participants understanding of each test was confirmed, the timer was set to 90 s and the participant would begin recall. Following Dritschel et al. (1992), subjects recalled information from the earlier autobiographical period first. Subsequent to the recall of each type of memory, there was a short pause of a few minutes, in which the experimenter prepared for the next phase and conversed with the subject at a general level. Following this, the next cycle of eye movements and testing began. The order in which episodic autobiographical, semantic, autobiographical (friends and teachers names), and general semantic memories were tested was counterbalanced. Thus the experiment consisted of a series of four eye movement and recall phases.

Following completion of the experiment, participants were debriefed and informed of their participant rights.

## Results

The number of memories recalled (minus repetitions or irrelevant information) for each memory type were entered into ANOVAs with eye movements as a between-subjects factor and autobiographical memory period and time period as within-subject factors. For episodic, friends and teacher memories this created the following design: three (eye movement; bilateral vs. vertical vs. central fixation) by two (autobiographical memory period; 5–11 vs. 12–18.) by three (recall period; 30 vs. 60 vs. 90) mixed ANOVA. For general semantic memory, the variable of autobiographical memory period was not relevant and the design was a three (eye movement; bilateral vs. horizontal vs. central fixation) by two (semantic category; animals vs. vegetables) by three (recall period; 30 vs. 60 vs. 90) mixed ANOVA.

### Episodic autobiographical memory

The cumulative number of specific autobiographical memories can be found in Table 1 below.

**Table 1:** **Mean (SD) number of episodic memories recalled as a function of eye movement condition, autobiographical memory period, and recall period**.

ABM period and recall period (s)	Eye movement		
	Bilateral	Vertical	Central
**5–11 YEARS**			
30	6.09 (3.36)	4.91 (2.15)	4.52 (1.44)
60	10.00 (4.22)	9.00 (3.87)	8.00 (2.43)
90	13.73 (4.78)	12.13 (4.69)	11.00 (3.50)
**12–18 YEARS**			
30	6.65 (2.53)	6.00 (2.45)	4.91 (2.69)
60	11.13 (3.25)	10.01 (3.25)	8.48 (3.63)
90	15.08 (4.19)	13.26 (3.73)	11.52 (4.44)

This revealed a main effect of autobiographical memory period, *F*(1, 66) = 7.53, *p* = 0.008, indicating more episodic memories recalled for the more recent period (*M* for 5–11 = 8.21; *M* for 12–18 = 9.67). The interaction between autobiographical memory period and eye movements was not significant *F*(2, 66) = 0.39, *p* = 0.68. The main effect of recall period was significant, *F*(2, 132) = 564.56, *p* ≤ 0.001, indicating a greater number of memories recalled over the longer (90 s) time period. The interaction between recall period and eye movements started to approach significance, *F*(4, 132) = 2.07, *p* = 0.09. The three way interaction between autobiographical memory period, recall period, and eye movement was not significant, *F*(4, 132) = 0.87, *p* = 0.42. The main effect of eye movements was significant. *F*(2, 66) = 3.57, *p* = 0.03. The main effect of eye movements was assessed via t tests between each of the eye movement conditions. The difference between the central and vertical condition started to approach significance, *t*(44) = −1.35, *p* = 0.09 (with higher means for the vertical condition). The difference between vertical and bilateral condition also started to approach significance, *t*(44) = −1.33, *p* = 0.09 (higher means for the bilateral condition). The difference between the central and bilateral condition was significant, *t*(44) = −2.66, *p* = 0.01 (with higher means for the bilateral condition).

### Semantic autobiographical memory – friends

The cumulative number of friends recalled can be found in Table 2 below.

**Table 2:** **Mean (SD) number of friend memories recalled as a function of eye movement condition, autobiographical memory period, and recall period**.

ABM period and recall (s)	Eye movement		
	Bilateral	Vertical	Central
**5–11 YEARS**			
30	11.91 (2.89)	11.52 (3.55)	9.95 (4.64)
60	18.30 (3.53)	17.69 (5.41)	16.39 (8.87)
90	22.17 (6.29)	21.17 (6.56)	20.83 (10.60)
**12–18 YEARS**			
30	13.43 (2.50)	12.65 (3.46)	10.65 (3.58)
60	21.48 (4.88)	20.08 (4.74)	17.65 (6.55)
90	27.56 (8.08)	25.73 (5.32)	23.09 (9.46)

The main effect of autobiographical memory period was significant *F*(1, 66) = 16.43, *p* ≤ 0.01, indicating more friend memories recalled for the more recent time period (*M* for 5–11 = 16.62; *M* for 12–18 = 19.15). The interaction between autobiographical memory period and eye movements was not significant, *F*(2, 66) = 0.88, *p* = 0.42. The main effect of recall period was significant, *F*(2, 132) = 374.91, *p* ≤ 0.01, indicating a greater number of friend memories recalled over the longer (90 s) time period. The interaction between recall period and eye movement was not significant *F*(4, 132) = 0.17, *p* = 0.94. The interaction between autobiographical memory period and recall period was significant, *F*(2, 132) = 10.37, *p* ≤ 0.001. This interaction was further assessed with simple main effects at each level of recall period. At the 30 s interval, the difference between 5–11 and 12–18 was significant, *t*(69) = −2.70, *p* = 0.009. At the 60 s interval, the difference between 5–11 and 12–18 was also significant, *t*(69) = −3.46, *p* = 0.001. At the 90 s interval, the difference between 5–11 and 12–18 was also significant, *t*(69) = −4.19, *p* ≤ 0.001. Overall, this indicates that the difference in the number of memories recalled between time periods concerning friends becomes more significant over the recall interval. The three way interaction between time period, time interval, and eye movement was not significant, *F*(4, 132) = 0.59, *p* = 0.67. The main effect of eye movements was not significant. *F*(2, 66) = 1.94, *p* = 0.15.

### Semantic autobiographical memory – teachers

The cumulative number of teachers recalled can be found in Table 3 below.

**Table 3:** **Mean (SD) number of teacher memories recalled as a function of eye movement condition, autobiographical memory period, and recall period**.

ABM period and recall period (s)	Eye movement		
	Bilateral	Vertical	Central
**5–11 YEARS**			
30	5.26 (1.73)	5.39 (2.77)	6.17 (3.47)
60	7.17 (2.46)	7.43 (3.59)	9.00 (6.78)
90	8.26 (2.63)	8.47 (3.83)	10.47 (6.85)
**12–18 YEARS**			
30	6.47 (2.57)	7.08 (2.87)	6.26 (4.47)
60	10.56 (3.69)	11.13 (4.29)	10.70 (7.60)
90	13.73 (4.85)	13.91 (4.32)	13.70 (9.56)

The main effect of autobiographical memory period was significant *F*(1, 66) = 25.69, *p* ≤ 0.001, indicating more teacher memories recalled for the more recent time period (*M* for 5–11 = 7.51; *M* for 12–18 = 10.40). The interaction between autobiographical memory period and eye movements was not significant *F*(2, 66) = 1.15, *p* = 0.32. The main effect of recall period was significant, *F*(2, 132) = 165.87, *p* ≤ 0.001, indicating a greater number of memories recalled over the longer (90 s) time period. The interaction between recall period and eye movements was not significant, *F*(4, 132) = 0.50, *p* = 0.74. The interaction between memory period and recall period was significant *F*(2, 132) = 29.83, *p* ≤ 0.001. The interaction was assessed with simple main effects at each level of time interval. At the 30 s interval, the difference between 5–11 and 12–18 was significant, *t*(69) = −2.43, *p* = 0.018. At the 60 s interval, the difference between 5–11 and 12–18 was also significant, *t*(69) = −4.85, *p* ≤ 0.001. At the 90 s interval, the difference between 5–11 and 12–18 was also significant, *t*(69) = −5.78, *p* ≤ 0.001. Overall, this indicates that the difference in the number of memories recalled between time periods concerning teachers becomes more significant over the recall interval. The three way interaction between memory period, recall period and eye movement was not significant, *F*(4, 132) = 0.23, *p* = 0.91. The main effect of eye movements was not significant. *F*(2, 66) = 0.27, *p* = 0.77.

### General semantic memory

The cumulative number of semantic items recalled can be found in Table 4 below.

**Table 4:** **Mean (SD) number of semantic memories recalled as a function of eye movement condition, semantic category, and recall period**.

Semantic category and recall period (s)	Eye movement		
	Bilateral	Vertical	Central
**VEGETABLES**			
30	9.26 (2.70)	9.09 (3.17)	8.87 (2.83)
60	13.47 (4.71)	12.43 (3.53)	13.35 (4.10)
90	16.00 (4.76)	14.56 (4.25)	15.52 (5.08)
**ANIMALS**			
30	15.91 (5.45)	14.39 (3.49)	15.09 (2.90)
60	24.78 (6.34)	22.26 (4.23)	23.70 (5.57)
90	29.96 (6.45)	28.08 (5.12)	29.47 (6.80)

The main effect of semantic category was significant *F*(1, 66) = 375.48, *p* ≤ 0.001. This indicates more items recalled for the category of animals (*M* for animals = 22.63; *M* for vegetables = 12.51). The interaction between semantic category and eye movements was not significant *F*(2, 66) = 0.36, *p* = 0.70. The main effect of recall period was significant, *F*(2, 132) = 488.94, *p* ≤ 0.001, indicating a greater number of exemplars recalled over the longer (90 s) time period. The interaction between recall period and eye movements was not significant, *F*(4, 132) = 0.57, *p* = 0.68. The interaction of semantic category and recall period interval was significant, *F*(2, 132) = 106.84, *p* ≤ 0.001. The interaction between semantic category and time interval was further assessed with simple main effects at each level of recall period. At the 30 s interval, the difference between vegetables and animals was significant, *t*(69) = −12.72, *p* ≤ 0.001. At the 60 s interval, the difference between vegetables and animals was also significant, *t*(69) = −18.51, *p* ≤ 0.001. At the 90 s interval, the difference between vegetables and animals was also significant, *t*(69) = −18.93, *p* ≤ 0.001. Overall, this indicates that the difference in the number of memories recalled between semantic categories periods becomes more significant over the recall interval. The three way interaction between semantic category, recall period and eye movements was not significant, *F*(4, 132) = 0.24, *p* = 0.91. The main effect of eye movement was not significant, *F*(2, 66) = 0.90, *p* = 0.41.

### General summary

Bilateral saccades enhanced autobiographical memory fluency but only when this required the retrieval of episodic information. Bilateral eye movements did not influence fluency on the tests requiring the retrieval of personals semantic information or taxonomic semantic information.

## Discussion

The current experiment found a dissociation between episodic and semantic autobiographical memory as a function of bilateral saccadic eye movements. A dissociation was also found between episodic autobiographical memory and general semantic memory. In particular, saccadic bilateral eye movements improved fluency of memory retrieval but only for episodic memory.

These findings are congruent with the explanation offered by Christman et al. (2003) in which it is claimed that bilateral saccades influence only episodic memory. The rationale for this was rooted in research based on the HERA model (Habib et al., 2003) which states episodic memory is dependent upon the efficient interaction between the two cerebral hemispheres. Consequently, one way to explain the current findings is that increased/more efficient hemispheric interaction brought about by bilateral saccades enabled the more effective recovery of episodic (vs. semantic) memory traces.

An interesting point is that the autobiographical memory period did not interact with the eye movement manipulation; the effects were observed irrespective of whether the memories were more recent or more remote. Earlier work has demonstrated that bilateral saccades can enhance the recovery of earlier autobiographical memories, and hence lead to an earlier offset of infantile amnesia (Christman et al., 2006). However, that experiment did not specifically compare different time periods. Furthermore, in the current experiment, although lifetime periods were specified, the experimental instructions did not request specifically the retrieval of earliest memories.

The interaction between eye movements and recall period did not reach statistical significance therefore, eye movements enabled the more fluent recovery of episodic information over the three recall periods (30, 60, and 90 s). However, although not significant, the interaction between recall period and eye movements started to approach such a value. Inspection of the means in Table 1 appears to show greater differences between the eye movement conditions for the longest recall period of 90 s. This may suggest that the effects of eye movements on recall could be greater when extended searches are required. This conjecture obviously requires further work.

The explanation for the effects of eye movements, as noted earlier, is based upon the original one formulated by Christman and colleagues (e.g., Christman et al., 2003; Christman and Propper, 2010). In some sense, direct evidence for the influence of bilateral saccades on hemispheric interaction has yet to found. Some suggestive evidence has shown that bilateral saccades alter prefrontal EEG coherence in the Gamma frequency range (Propper et al., 2007). However, this experiment did not measure memory performance. In another study, that did measure memory, no evidence was found for a specific effect of bilateral saccades on hemispheric interaction even though memory was improved following bilateral eye movements (Samara et al., 2011).

An alternative account of the results is that eye movements may enhance anterior-posterior interactions (Parker and Dagnall, 2007). Such interactions are of particular importance for episodic memory retrieval (Simons and Spiers, 2003; Summerfield and Mangels, 2005), and have been taken to indicate the functional coupling of frontal executive processes with more posterior regions, where the memory trace is stored. More recently, Lyle and colleagues (e.g., Lyle and Martin, 2010; Lyle and Orsborn, 2011) hypothesize that saccadic eye movements may result in the activation of neural regions that are involved in the allocation of attention and top-down control mechanisms. Their explanation is based on the findings that show making saccades activates a network of neural regions that include the frontal eye fields and more posterior regions such as the intraparietal sulcus. The latter region has been shown to play a role in episodic memory (Skinner and Fernandes, 2007; Cabeza, 2008; Ciaramelli et al., 2008) and the idea is that saccadic eye movements serve to pre-activate this region and thus enhance its functional contributions to task performance. These anterior-posterior interactions can occur *within* each hemisphere and thus in some respects, does not require interhemispheric interaction in order to support memory. Evidence for this has come from behavioral studies in which bilateral saccades were found to increase the accuracy of detection in a letter matching task, but only for within hemisphere trials (Lyle and Martin, 2010). Support has also been found from findings that demonstrate saccades do not increase effects that depends on interhemispheric interaction such as bilateral gain effects in face recognition (Lyle and Orsborn, 2011). More direct evidence for the role of saccade execution in attention control comes from Edlin and Lyle (2013), who found that bilateral saccades enhanced performance in the Attentional Network Task (ANT). In this task, subjects are required to detect and indicate the direction in which a target arrow is pointing. The position of the arrow can be cued (vs. uncued) and can be flanked by arrows that are either congruent (vs. incongruent) with the targets direction. The ANT allows for an assessment of various forms of attentional operations including, alerting, orienting, and control (executive functioning). Their results indicated an effect of bilateral saccades on the executive control component.

In relation to the current findings, the notion of enhanced top-down control and anterior-posterior interactions would make sense from some theoretical models of autobiographical memory and neuroimaging findings. For example, Conway (2001, 2005) and Conway and Pleydell-Pearce (2000), conceptualize autobiographical memory as organized in a hierarchical manner with distinct but interacting levels of representations. At the highest level are lifetime periods which represent personal information covering broad spans of time. Examples could include “my life whilst at secondary school” or “my relationship with my partner.” Also included at this level is general knowledge of other persons (e.g., teachers and friends), actions and plans. This type of representation is devoid of event-specific information and can be considered more schematic or semantic in nature. The next level of representation refers to general events and refers to both repeated and extended events such as “my holiday in the Lake District.” The time span for this level typically ranges from days to weeks. The most detailed level of representation is that of event-specific knowledge. This level contains information of specific experiences rooted in perception and linked to occurrences in time and place. Consequently, this level of representation is episodic in nature.

Recalling autobiographical information can take the form of accessing event-specific information via higher levels of representation via control processes. Thus, recalling semantic information takes place prior to accessing the episodic details of an event. In relation to neural activations, this translates into initial processing in anterior regions, such as the left prefrontal cortex (representing cue specification and elaboration) followed by activations in posterior regions on the right or bilaterally (as the memory is recovered). This idea has received empirical support from studies of both slow cortical EEG potentials (e.g., Conway et al, 2001) and fMRI research (e.g., Botzung et al., 2008) (see also Cabeza and St Jacques, 2007 for a review of imaging studies implicating anterior-posterior interactions). More generally, a role for executive functioning in retrieving autobiographical memories has been implicated across a range of work, especially when access is required to event or episode-specific information. This is demonstrated by reduced executive control being associated with the increased likelihood of recalling more general (less specific) memories (e.g., Dalgleish et al., 2007), fewer episodic details (e.g., Matuszewski et al., 2009), and a reduced sense of autonoetic awareness and reliving of the episode (e.g., Piolino et al., 2007).

In terms of the present findings, this would suggest that bilateral saccades facilitate access to lower levels of representation. Higher levels of representation (semantic memory) are not influenced by such eye movements. Consequently, an alternative explanation of the current findings is that the dissociation between the retrieval of event-specific (episodic) and more general semantic memory arises as a direct result of top-down executive mechanisms as specified by Edlin and Lyle (2013). This would presumably take the form of saccades enhancing executive functioning, preactivating posterior regions involved in episodic memory, and enabling a more efficient and extended search through the store of event-specific information.

The idea of the importance of anterior-posterior interaction in autobiographical retrieval (and eye movement effects) does not have to be seen as excluding hemispheric interactions. Cooperation between the hemispheres would appear to be important in recalling episodic autobiographical memory as indicated by imaging studies that find bilateral activations during recall (e.g., Markowitsch et al., 2000; Greenberg et al., 2005; Vandekerckhove et al., 2005; Viard et al., 2010; Söderlund et al., 2012). Conceivably therefore, both accounts of eye movement effects could provide a plausible account of the findings; the experiment itself was not designed to assess one compared to the other.

Some of the findings of the present experiment deserve further comments; in particular, as related to recall period (and the duration of eye movement effects) and vertical saccades. Although the effect of eye movements did not interact with recall period (or ABM period), consideration of the means for episodic memory, appear to show slightly larger differences for the later segment of the recall period. Indeed the interaction started to approach significance for these variables. Whilst it is difficult to make too much of this finding, it is interesting to speculate why this might occur in relation to; (i) the potential time-limited effect of eye movements, and (ii) the means by which eye movements influence episodic retrieval. Pertaining to the former, the influence of eye movements must have at least spanned the recall period and thus show effects lasting over 180 s. This would certainly be within the potential time-window for eye movement effects as noted in the introduction. Pertaining to the latter, if it is assumed that recall in autobiographical memory may often start with the recall of the most accessible information or spontaneously retrieved memories (e.g., Conway and Pleydell-Pearce, 2000), then eye movements do not appear to influence this early stage of retrieval. Instead, the largest influence could arise at a later stage in the recall process that represents more effortful or attentionally demanding processing. This form of retrieval requires a generative recall process likely involving frontal/executive processes (Smith et al., 2010). This finding, if robust, fits in well with the hypothesis that saccade execution influences effortful recall by the implementation of top-down control processes.

It was noted in the introduction that only bilateral saccades were expected to enhance memory as this is in accordance with the idea that such influences are underpinned by hemispheric interaction. However, some research has found vertical saccades to enhance episodic memory (e.g., Lyle et al., 2008a), whereas other research has not found this to be the case (e.g., Christman et al., 2003; Parker et al., 2008; Brunyé et al., 2009). The current research found that vertical eye movements produced effects that were in-between those of bilateral and the central fixation condition (see Table 1). In the original paper by Christman et al. (2003), vertical eye movements (and other forms of eye movements) produced effects that were in-between the bilateral and no-eye movement condition. It is unclear why such differences have been observed in previous work and suggests additional research is required.

There are some limitations to the current experiment that need to be considered. One relates to the study of autobiographical memory more generally. In particular, because the events sampled come from the participants own life, then it is difficult to independently assess the veracity of the information recalled. One potential means of overcoming this would be to use diary based methods (e.g., Christman et al., 2003), but this would typically limit the time period over which memory could be studied. In relation to much autobiographical memory work that uses fluency measure or other forms of retrospective accounts (e.g., cue-word techniques), then it needs to be ensured that recalls are not contaminated by false memories, misleading reports (perhaps because of personal details), or retrieval from incorrect time periods. In the current experiment, the participants were fully assured that all responses would be treated anonymously and that they were not pressured into producing any personal details that they felt uncomfortable revealing. It would also seem unlikely that the results obtained here are due to any unintentional production of false memories or some form of source monitoring error by identifying memories from incorrect time periods. This is argued because previous laboratory-based work on bilateral eye movements has shown them to increase true and decrease false memory, even in situations that require a high degree of source monitoring to reduce such errors (e.g., Parker et al., 2008; Lyle and Jacobs, 2010). Consequently, to contend that the effects that were found here were the result of false autobiographical recall or faulty source monitoring would be incongruent with past research.

Another potential limitation is that handedness was not assessed. In some experiments, handedness and eye movements have been shown to interact such that greater eye movement effects are found with individuals who are strongly right-handed (Lyle et al., 2008b; Brunyé et al., 2009). However, other work has shown eye movements to enhance memory irrespective of handedness (Lyle and Jacobs, 2010). More recently, consistency of handedness (regardless of direction) has been shown to be of importance (Lyle et al., 2012). As a result, the precise relationship between handedness and eye movements is something that has yet to be explored further, both in general terms and in more specific contexts like the experiment reported here. In spite of this, the fact that an effect of bilateral eye movement was found suggests that even if stronger effects can be found with right-handed participants, they are not negligible in groups that likely contain a broader range of handedness types. In addition, given that most common behavioral profile is that of right-handedness (Peters and Murphy, 1992), then it could be safe to assume that most of the participants in the current experiment were right-handed.

Handedness itself has an influence on cognitive processes as outlined in the introduction; for example enhancing episodic memory. As some research has indicated similar effects of both eye movements and handedness (see Christman and Propper, 2010 for a review), then one might expect similar outcomes to the present results when comparing inconsistent with consistently strong right-handers. Although this research has not been undertaken, one recent report showed that general semantic memory fluency (number of exemplars produced) was not influenced by handedness (Sontam et al., 2009), and is thus consistent with the findings here. However, they did find a difference in which mixed-handed subjects demonstrated greater semantic switching (shifting from one taxonomic category to another) which was taken to indicate more widespread activation within semantic networks.

In summary, the present results found a dissociation between the episodic and semantic components of autobiographical memory as a function of eye movements. Subsequent research may consider investigating the influence of eye movements on other tasks of autobiographical memory and examining in a more direct manner, the influence of hemispheric interaction on both episodic and semantic autobiographical retrieval.

## Conflict of Interest Statement

The authors declare that the research was conducted in the absence of any commercial or financial relationships that could be construed as a potential conflict of interest

## References

[B1] Babiloni C. Babiloni F. Carducci F. Cappa S. Cincotti F. Del Percio C. (2004). Human cortical EEG rhythms during long-term episodic memory task. A high-resolution EEG study of the HERA model. Neuroimage 21, 1576–1584 10.1016/j.neuroimage.2003.11.023 15050581

[B2] Babiloni C. Vecchio F. Cappa S. Pasqualetti P. Rossi S. Miniussi C. (2006). Functional frontoparietal connectivity during encoding and retrieval processes follows the HERA model: a high-resolution study. Brain Res. Bull. 68, 203–212 10.1016/j.brainresbull.2005.04.019 16377425

[B3] Blum S. Yonelinas A. P. (2001). Transfer across modality in perceptual implicit memory. Psychon. Bull. Rev. 8, 147–154 11340860 10.3758/bf03196151

[B4] Botzung A. Denkova E. Ciuciu P. Scheiber C. Manning L. (2008). The neural bases of the constructive nature of autobiographical memories studied with a self-paced fMRI design. Memory 16, 351–365 10.1080/09658210801931222 18432480

[B5] Brunyé T. T. Mahoney C. R. Augustyn J. S. Taylor H. A. (2009). Horizontal saccadic eye movements enhance the retrieval of landmark shape and location information. Brain Cogn. 70, 279–288 10.1016/j.bandc.2009.03.003 19346050

[B6] Cabeza R. (2008). Role of parietal regions in episodic memory retrieval: the dual attentional processes hypothesis. Neuropsychologia 46, 1813–1827 10.1016/j.neuropsychologia.2008.03.019 18439631 PMC2517132

[B7] Cabeza R. St Jacques P. (2007). Functional neuroimaging of autobiographical memory. Trends Cogn. Sci. 11, 219–227 10.1016/j.tics.2007.02.005 17382578

[B8] Christman S. (1993). Handedness in musicians: bimanual constraints upon performance. Brain Cogn. 22, 266–272 8373577 10.1006/brcg.1993.1038

[B9] Christman S. (1995). “Independence versus integration of right and left hemisphere processing: effects of handedness,” in Hemispheric Communication: Mechanisms and Models, ed. Kitterle F. L. (Hillsdale, NJ: Erlbaum), 231–253

[B10] Christman S. D. Butler M. (2011). Mixed-handedness advantages in episodic memory obtained under conditions of intentional learning extend to incidental learning. Brain Cogn. 77, 17–22 10.1016/j.bandc.2011.07.003 21807450

[B11] Christman S. D. Garvey K. J. Propper R. E. Phaneuf K. A. (2003). Bilateral eye movements enhance the retrieval of episodic memories. Neuropsychology 17, 221–229 10.1037/0894-4105.17.2.221 12803427

[B12] Christman S. D. Propper R. E. (2010). “Episodic memory and hemispheric interaction: handedness and eye movements,” in Current Issues in Applied Memory Research, eds Davies G. M. Wright D. B. (Hove: Psychology Press), 185–205

[B13] Christman S. D. Propper R. E. Brown T. J. (2006). Increased interhemispheric interaction is associated with earlier offset of childhood amnesia. Neuropsychology 20, 336–345 10.1037/0894-4105.20.3.336 16719626

[B14] Christman S. D. Propper R. E. Dion A. (2004). Increased interhemispheric interaction is associated with deceased false memories in a verbal converging semantic associates paradigm. Brain Cogn. 56, 313–319 15522769 10.1016/j.bandc.2004.08.005

[B15] Chu O. Abeare C. A. Bondy M. A. (2012). Inconsistent vs consistent right-handers’ performance on an episodic memory task: evidence from the California Verbal learning Test. Laterality 17, 306–317 10.1080/1357650X.2011.568490 22594813

[B16] Ciaramelli E. Grady C. L. Moscovitch M. (2008). Top-down and bottom-up attention to memory: a hypothesis (AtoM) on the role of the posterior parietal cortex in memory retrieval. Neuropsychologia 46, 1828–1851 10.1016/j.neuropsychologia.2008.03.022 18471837

[B17] Clarke J. M. Zaidel E. (1994). Anatomical-behavioral relationships: corpus callosum morphometry and hemispheric specialization. Behav. Brain Res. 64, 185–202 10.1016/0166-4328(94)90131-7 7840886

[B18] Conway M. A. (1990). Autobiographical Memory: An Introduction. Buckingham: Open University Press

[B19] Conway M. A. (2001). Sensory-perceptual episodic memory and its context: autobiographical memory. Philos. Trans. R. Soc. Lond. B. Biol. Sci. 356, 1375–1384 10.1098/rstb.2001.0940 11571029 PMC1088521

[B20] Conway M. A. (2005). Memory & the self. J. Mem. Lang. 53, 594–628

[B21] Conway M. A. Pleydell-Pearce C. W. (2000). The construction of autobiographical memories in the self-memory system. Psychol. Rev. 107, 261–288 10.1037/0033-295X.107.2.261 10789197

[B22] Conway M. A. Pleydell-Pearce C. W. Whitecross S. E. (2001). The neuroanatomy of autobiographical memory: A slow cortical potential study of autobiographical memory retrieval. J. Mem. Lang. 45, 493–524 10.1006/jmla.2001.2781

[B23] Coste C. Agar N. Petitfour E. Quinette P. Guillery-Girard B. Azouvi P. (2011). Exploring the roles of the executive and short-term feature binding functions in the retrieval of retrograde autobiographical memories in severe traumatic brain injury. Cortex 47, 771–786 10.1016/j.cortex.2010.07.004 20817159

[B24] Cronin-Golomb A. Gabrieli J. D. E. Keane M. M. (1996). Implicit & explicit memory retrieval within and across the disconnected cerebral hemispheres. Neuropsychology 10, 254–262

[B25] Dalgleish T. Williams J. M. Golden A. M. Perkins N. Barrett L. F. Barnard P. J. (2007). Reduced specificity of autobiographical memory and depression: the role of executive control. J. Exp. Psychol. Gen. 136, 23–42 10.1037/0096-3445.136.1.23 17324083 PMC2225543

[B26] Dean H. L. Crowley J. C. Platt M. L. (2004). Visual and saccade-related activity in macaque posterior cingulate cortex. J. Neurophysiol. 92, 3056–3068 10.1152/jn.00691.2003 15201314

[B27] Denenberg V. H. Kertesz A. Cowell P. E. (1991). A factor analysis of the human’s corpus callosum. Brain Res. 548, 126–132 10.1016/0006-8993(91)91113-F 1907877

[B28] Dritschel B. H. Williams J. M. G. Baddeley A. D. Nimmo-Smith I. (1992). Autobiographical fluency – a method for the study of personal memory. Mem. Cognit. 20, 133–140 1565011 10.3758/bf03197162

[B29] Düzel E. Cabeza R. Pictons T. W. Yonelinas A. P. Scheich H. Heinze H.-J. (1999). Task-related and item-related brain processes of memory retrieval. Proc. Natl. Acad. Sci. U.S.A. 96, 1794–1799 10.1073/pnas.96.4.1794 9990104 PMC15598

[B30] Edlin J. M. Lyle K. B. (2013). The effect of repetitive saccade execution on the attention network test: enhancing executive function with a flick of the eyes. Brain Cogn. 81, 345–351 10.1016/j.bandc.2012.12.006 23485024

[B31] Gagnon G. Blanchet S. Grondin S. Schneider C. (2010). Paired-pulse transcranial magnetic stimulation over the dorsolateral prefrontal cortex interferes with episodic encoding and retrieval for both verbal and non-verbal materials. Brain Res. 1344, 148–158 10.1016/j.brainres.2010.04.041 20462501

[B32] Geffen G. M. Forrester G. M. Jones D. L. Simpson D. A. (1994). “Auditory verbal learning and memory in cases of callosal agenesis,” in Callosal Agenesis, eds Lassonde M. Jeeves M. A. (New York, NY: Plenum Press), 247–260

[B33] Gilboa A. Ramirez J. Kohler S. Westmacott R. Black S. E. Moscovitch M. (2005). Retrieval of autobiographical memory in Alzheimer’s disease: relation to volumes of medial temporal lobe and other structures. Hippocampus 15, 535–550 10.1002/hipo.20090 15884035

[B34] Greenberg D. L. Rice H. J. Cooper J. J. Cabeza R. Rubin D. C. LaBar K. S. (2005). Co-activation of the amygdala, hippocampus and inferior frontal gyrus during autobiographical memory retrieval. Neuropsychologia 43, 659–674 10.1016/j.neuropsychologia.2004.09.002 15721179

[B35] Greene J. D. Hodges J. R. Baddeley A. D. (1995). Autobiographical memory and executive function in early dementia of Alzheimer’s type. Neuropsychologia 33, 1647–1670 10.1016/0028-3932(95)00046-1 8745122

[B36] Habib R. Gayraud D. Oliva A. Regis J. Salamon G. Khalil R. (1991). Effects of handedness and sex on the morphology of the corpus callosum: a study with brain magnetic resonance imaging. Brain Cogn 16, 41–61 1854469 10.1016/0278-2626(91)90084-l

[B37] Habib R. Nyberg L. Tulving E. (2003). Hemispheric asymmetries of memory: the HERA model revisited. Trends Cogn. Sci. 7, 241–245 10.1016/S1364-6613(03)00110-4 12804689

[B38] Jha A. P. Kroll N. Baynes K. Gazzaniga M. S. (1997). Memory encoding following complete commissurotomy. J. Cogn. Neurosci. 9, 143–159 10.1162/jocn.1997.9.1.143 23968186

[B39] Kastner S. DeSimone K. Konen C. S. Szczepanski S. Weiner K. S. Schneider K. A. (2007). Topographic maps in human frontal cortex revealed in delayed saccade and spatial working memory tasks. J. Neurophysiol. 97, 3494–3507 10.1152/jn.00010.2007 17360822

[B40] Kempe V. Brooks P. J. Christman S. D. (2009). Inconsistent handedness is linked to more successful foreign language vocabulary learning. Psychon. Bull. Rev. 16, 480–485 10.3758/PBR.16.3.480 19451372

[B41] Kompus K. Kalpouzos G. Westerhausen R. (2011). The size of the anterior corpus callosum correlates with the strength of hemispheric encoding-retrieval asymmetry in the ventrolateral prefrontal cortex. Brain Res. 1419, 61–67 10.1016/j.brainres.2011.08.052 21925652

[B42] Kopelman M. D. Wilson B. A. Baddeley A. D. (1989). The autobiographical memory interview: a new assessment of autobiographical and personal semantic memory in amnesic patients. J. Clin. Exp. Neuropsychol. 11, 724–744 10.1080/01688638908400928 2808661

[B43] Levine B. (2004). Autobiographical memory and the self in time: brain lesion effects, functional neuroanatomy, and lifespan development. Brain Cogn. 55, 54–68 15134843 10.1016/S0278-2626(03)00280-X

[B44] Levine B. Svoboda E. Hay J. F. Winocur G. Moscovitch M. (2002). Aging and autobiographical memory: dissociating episodic from semantic retrieval. Psychol. Aging 17, 677–689 10.1037/0882-7974.17.4.677 12507363

[B45] Lyle K. B. Hanaver-Torrez S. D. Hackländer R. P. Edlin J. M. (2012). Consistency of handedness regardless of direction predicts baseline memory accuracy and potential for memory enhancement. J. Exp. Psychol. Learn. Mem. Cogn. 38, 187–193 10.1037/a0024831 21843021

[B46] Lyle K. B. Jacobs N. (2010). Is saccade-induced retrieval enhancement a potential means of improving eyewitness evidence? Memory 18, 581–594 10.1080/09658211.2010.493891 20658433

[B47] Lyle K. B. Logan J. M. Roediger H. L. (2008a). Eye movements enhance memory for individuals who are strongly right-handed and harm it for individuals who are not. Psychon. Bull. Rev. 15, 515–520 18567248 10.3758/pbr.15.3.515

[B48] Lyle K. B. McCabe D. P. Roediger H. L. (2008b). Handedness is related to memory via hemispheric interaction: evidence from paired associate recall and source memory tasks. Neuropsychology 22, 523–530 10.1037/0894-4105.22.4.523 18590363

[B49] Lyle K. B. Martin J. M. (2010). Bilateral saccades increase intrahemispheric processing but not interhemispheric interaction: implications for saccade-induced retrieval enhancement. Brain Cogn. 73, 128–134 10.1016/j.bandc.2010.04.004 20452714

[B50] Lyle K. B. Orsborn A. E. (2011). Inconsistent handedness and saccade execution benefit face memory without affecting interhemispheric interaction. Memory 19, 613–624 10.1080/09658211.2011.595418 21919589

[B51] Manenti R. Cotelli M. Miniussi C. (2011). Successful physiological aging and episodic memory: a brain stimulation study. Behav. Brain Res. 216, 153–158 10.1016/j.bbr.2010.07.027 20667492

[B52] Markowitsch H. J. Theiel A. Reinkemeier M. Kessler J. Koyuncu A. Heiss W. D. (2000). Right amygdalar and temporofrontal activation during autobiographic, but not during fictitious memory retrieval. Behav. Neurol. 12, 181–190 11568430 10.1155/2000/303651

[B53] Matuszewski V. Piolino P. Belliard S. de la Sayette V. Laisney M. Lalevee C. (2009). Patterns of autobiographical memory impairment according to disease severity in semantic dementia. Cortex 45, 456–472 10.1016/j.cortex.2007.11.006 19231476

[B54] McDermott K. B. Ojemann J. G. Petersen S. E. Ollinger J. M. Snyder A. Z. Akbudak E. (1999). Direct comparison of episodic encoding and retrieval of words: an event-related fMRI study. Memory 7, 661–678 10.1080/096582199387797 10659091

[B55] Miller M. B. Kingstone A. Gazzaniga M. S. (2002). Hemispheric encoding asymmetry is more apparent than real. J. Cogn. Neurosci. 14, 702–708 10.1162/08989290260138609 12167255

[B56] Murphy K. J. Troyer A. K. Levine B. Moscovitch M. (2008). Episodic, but not semantic, autobiographical memory is reduced in amnestic mild cognitive impairment. Neuropsychologia 46, 3116–3123 10.1016/j.neuropsychologia.2008.07.004 18675285 PMC2629588

[B57] Nelson D. L. Keelean P. D. Negrao M. (1989). Word-fragment cuing: the lexical search hypothesis. J. Exp. Psychol. Learn. Mem. Cogn. 15, 388–397 2524544 10.1037//0278-7393.15.3.388

[B58] Niebauer C. L. Aselage J. Schutte C. (2002). Interhemispheric interaction and consciousness: degree of handedness predicts the intensity of a sensory illusion. Laterality 7, 85–96 15513190 10.1080/13576500143000159

[B59] Nieuwenhuis S. Elzinga B. M. Ras P. Berends F. Duijs P. Samara Z. (2013). Bilateral saccadic eye movements and tactile stimulation, but not auditory stimulation, enhance memory retrieval. Brain Cogn. 81, 52–56 10.1016/j.bandc.2012.10.003 23174428

[B60] Nolde S. F. Johnson M. K. Raye C. L. (1998). The role of prefrontal cortex during tests of episodic memory. Trends Cogn. Sci. 2, 399–406 10.1016/S1364-6613(98)01233-9 21227255

[B61] Nyberg L. Cabeza R. Tulving E. (1996). PET studies of encoding and retrieval: the HERA model. Psychon. Bull. Rev. 3, 135–148 10.3758/BF03212412 24213861

[B62] Parker A. Buckley S. Dagnall N. (2009). Reduced misinformation effects following saccadic bilateral eye movements. Brain Cogn. 69, 89–97 10.1016/j.bandc.2008.05.009 18635303

[B63] Parker A. Dagnall N. (2007). Effects of bilateral eye movements on gist based false recognition in the DRM paradigm. Brain Cogn. 63, 221–225 17027132 10.1016/j.bandc.2006.08.005

[B64] Parker A. Dagnall N. (2010). Effects of handedness and saccadic bilateral eye movements on components of autobiographical recollection. Brain Cogn. 73, 93–101 10.1016/j.bandc.2010.03.005 20413200

[B65] Parker A. Relph S. Dagnall N. (2008). Effects of bilateral eye movements on the retrieval of item, associative and contextual information. Neuropsychology 22, 136–145 10.1037/0894-4105.22.1.136 18211163

[B66] Pasqual-Leone A. Valls-Solé J. Wassermann E. M. Hallett M. (1994). Responses to rapid-rate transcranial magnetic stimulation of the human motor cortex. Brain 117, 847–858 10.1093/brain/117.4.847 7922470

[B67] Peters M. Murphy K. (1992). Cluster analysis reveals at least three, and possibly five, distinct handedness groups. Neuropsychologia 30, 373–380 10.1016/0028-3932(92)90110-8 1603300

[B68] Phelps E. A. Hirst W. Gazzaniga M. S. (1991). Deficits in recall following partial and complete commissurotomy. Cereb. Cortex 1, 492–498 10.1093/cercor/1.6.492 1822754

[B69] Piolino P. Desgranges B. Benali K. Eustache F. (2002). Episodic and semantic remote autobiographical memory in ageing. Memory 10, 239–257 10.1080/09658210143000353 12097209

[B70] Piolino P. Desgranges B. Manning L. North P. Jokic F. Eustache F. (2007). Autobiographical memory and the sense of recollection and executive functions after closed head injury. Cortex 43, 176–195 10.1016/S0010-9452(08)70474-X 17405665

[B71] Propper R. E. Christman S. D. (2004). Mixed-versus strong right-handedness is associated with biases towards “remember” versus “know” judgements in recognition memory: role of interhemispheric interaction. Memory 12, 707–714 10.1080/09658210344000503 15724359

[B72] Propper R. E. Christman S. D. Phaneuf K. A. (2005). A mixed-handed advantage in episodic memory: a possible role of interhemispheric interaction. Mem. Cognit. 33, 751–757 16248339 10.3758/bf03195341

[B73] Propper R. E. Pierce J. Bellorado N. Geisler M. W. Christman S. D. (2007). Effect of bilateral eye movements on frontal interhemispheric gamma EEG coherence: implications for EMDR therapy. J. Nerv. Ment. Dis. 195, 785–788 10.1097/NMD.0b013e318142cf73 17984782

[B74] Richardson-Klavehn A. Gardiner J. M. (1998). Depth of processing effects on priming is stem completion: test of the voluntary-contamination, conceptual-processing and lexical-processing hypotheses. J. Exp. Psychol. Learn. Mem. Cogn. 24, 593–609 9606929 10.1037//0278-7393.24.3.593

[B75] Roediger H. L. Weldon M. S. Stadler M. L. Riegler G. L. (1992). Direct comparison of two implicit memory tests: word fragment completion and word stem completion. J. Exp. Psychol. Learn. Mem. Cogn. 18, 1251–1269 1447550 10.1037//0278-7393.18.6.1251

[B76] Rossi S. Miniussi C. Pasqualetti P. Babiloni C. Rossini P. M. Cappa S. F. (2004). Age-related functional changes of prefrontal cortex in long-term memory: a repetitive transcranial magnetic stimulation study. J. Neurosci. 24, 7939–7944 10.1523/JNEUROSCI.0703-04.2004 15356207 PMC6729939

[B77] Rossi S. Pasqualetti P. Zito G. Vecchio F. Cappa S. F. Miniussi C. (2006). Prefrontal and parietal cortex in human episodic memory: an interference study by repetitive transcranial magnetic stimulation. Eur. J. Neurosci. 23, 793–800 10.1111/j.1460-9568.2006.04600.x 16487159

[B78] Samara Z. Elzinga B. M. Slagter H. A. Nieuwenhuis S. (2011). Do horizontal saccadic eye movements increase interhemispheric coherence? Investigation of a hypothesized neural mechanism underlying EMDR. Front. Psychiatry 2:4 10.3389/fpsyt.2011.00004 21556274 PMC3089996

[B79] Shobe E. R. Ross N. M. Fleck J. I. (2009). Influence of handedness and bilateral eye movements on creativity. Brain Cogn. 71, 204–214 10.1016/j.bandc.2009.08.017 19800726

[B80] Simons J. S. Spiers H. J. (2003). Prefrontal and medial temporal lobe interactions in long-term memory. Nat. Rev. Neurosci. 4, 637–648 10.1038/nrn1178 12894239

[B81] Skinner E. I. Fernandes M. A. (2007). Neural correlates of recollection and familiarity: a review of neuroimaging and patient data. Neuropsychologia 45, 2163–2179 10.1016/j.neuropsychologia.2007.03.007 17445844

[B82] Smith S. J. Souchay C. Conway M. A. (2010). Overgeneral autobiographical memory in Parkinson’s disease. Cortex 46, 787–793 10.1016/j.cortex.2009.08.006 19781694

[B83] Söderlund H. Moscovitch M. Kumar N. Mandic M. Levine B. (2012). As time goes by: hippocampal connectivity changes with remoteness of autobiographical memory retrieval. Hippocampus 22, 670–679 10.1002/hipo.20927 21404363

[B84] Sontam V. Christman S. D. Jasper J. D. (2009). Individual differences in semantic switching flexibility: effects of handedness. J. Int. Neuropsychol. Soc. 15, 1023–1027 10.1017/S1355617709990440 19709453

[B85] Summerfield C. Mangels J. A. (2005). Functional coupling between frontal and parietal lobes during recognition memory. Neuroreport 16, 117–122 10.1097/00001756-200502080-00008 15671858

[B86] Tulving E. (2002). “Episodic memory and common sense: how far apart?” in Episodic Memory: New Directions in Research, eds Baddeley A. Conway M. Aggleton J. (Oxford: Oxford University Press), 269–287

[B87] Tulving E. Kapur S. Craik F. I. M. Markowitsch H. J. Houle S. (1994). Hemispheric encoding and retrieval asymmetry in episodic memory: positron emission tomography findings. Proc. Natl. Acad. Sci. U.S.A. 91, 2016–2020 10.1073/pnas.91.6.2016 8134342 PMC43300

[B88] Unsworth N. Spillers G. J. Brewer G. A. (2012). The role of working memory capacity in autobiographical retrieval: individual differences in strategic search. Memory 20, 167–176 10.1080/09658211.2011.651087 22273543

[B89] Vandekerckhove M. M. P. Markowitsch H. J. Mertens M. Woermann F. G. (2005). Bi-hemisperic engagement in the retrieval of autobiographical memories. Behav. Neurol. 16, 203–210 16518010 10.1155/2005/460745 PMC5478841

[B90] Viard A. Lebreton K. Chételat G. Desgranges B. Landeau B. Young A. (2010). Patterns of hippocampal-neocortical connectivity in the retrieval of autobiographical memories across the entire life-span of aged adults. Hippocampus 20, 153–165 10.1002/hipo.20601 19338022 PMC3021758

[B91] Wagner A. D. Poldrack R. A. Eldridge L. L. Desmond J. E. Glover G. H. Gabrieli J. D. (1998). Material-specific lateralization of prefrontal activation during episodic encoding and retrieval. Neuroreport 9, 3711–3717 10.1097/00001756-199811160-00026 9858384

[B92] Witelson S. (1985). The brain connection: the corpus callosum is larger in left-handers. Science 229, 655–668 10.1126/science.4023705 4023705

